# Malignant transformation of white sponge nevus: a case report of a novel keratin 4 mutation

**DOI:** 10.1186/s12903-024-04300-y

**Published:** 2024-05-21

**Authors:** Dan Liu, Tianyu Zhang, Hangfan Zhou, Chuanji Wu, Taiwen Li, Lu Jiang

**Affiliations:** State Key Laboratory of Oral Diseases, National Clinical Research Center for Oral Diseases, Research Unit of Oral Carcinogenesis and Management, Chinese Academy of Medical Sciences, West China Hospital of Stomatology, Sichuan University, Renmin Nan Street Section 3 NO 14, Chengdu, Sichuan 610041 P. R. China

**Keywords:** White sponge nevus, Keratin 4 gene, Genetic mutation, Malignant transformation, Single-cell RNA sequencing

## Abstract

**Background:**

White Sponge Nevus (WSN) is traditionally considered a benign genetic disorder affecting the oral mucosa, primarily caused by pathogenic mutations in keratin 4 (*KRT4*) or keratin 13 (*KRT13*). Despite its benign nature, recent evidence has begun to question the malignant potential of WSN.

**Case presentation:**

We report a case involving a 70-year-old man who presented with a white lesion on the right floor of his mouth. Initial diagnostic evaluations confirmed the lesion as WSN. Over a one-year follow-up, the lesion underwent malignant transformation, evolving into local epithelial moderate-to-severe dysplasia. Exome sequencing identified a novel insertion mutation in exon 1 of the *KRT4* gene, resulting in a deletion-insertion amino acid mutation involving glycine. Single-cell RNA sequencing further revealed altered epithelial proliferation and differentiation dynamics within the lesion.

**Conclusions:**

This case not only expands the known genetic spectrum of *KRT4* mutations associated with WSN but also provides preliminary evidence suggesting the malignant potential of WSN. The novel pathogenic mutation in *KRT4* is postulated to alter epithelial proliferation and differentiation, thereby raising concerns about the malignant transformation of WSN. Further studies are warranted to confirm these findings.

## Background

White Sponge Nevus (WSN) is a rare autosomal dominant disorder with a prevalence of less than 1:200,000. While the majority of cases are familial, a small proportion are sporadic, and no sex-based differences have been observed [1]. The disorder primarily manifests as symmetrical white spongy plaques on the nonkeratinized oral mucosa, although occurrences in extraoral mucosa have also been reported [2]. Pathogenic variants in keratin 4 (*KRT4*) or keratin 13 (*KRT13*) are often implicated in WSN, likely due to alterations in the structure and function of the encoded proteins [1, 3]. Traditionally, WSN has been considered a benign condition. However, recent case reports have indicated its potential for malignant transformation [4–6]. Understanding this malignant potential is critical for both patient management and prognosis. In light of this, we present a one-year follow-up case of a WSN lesion that underwent malignant transformation due to a novel *KRT4* gene mutation. This study aims to investigate the relationship between new *KRT4* mutations and the malignant transformation potential of WSN.

## Case presentation

### Clinical presentation and initial diagnosis

**Fig. 1:**
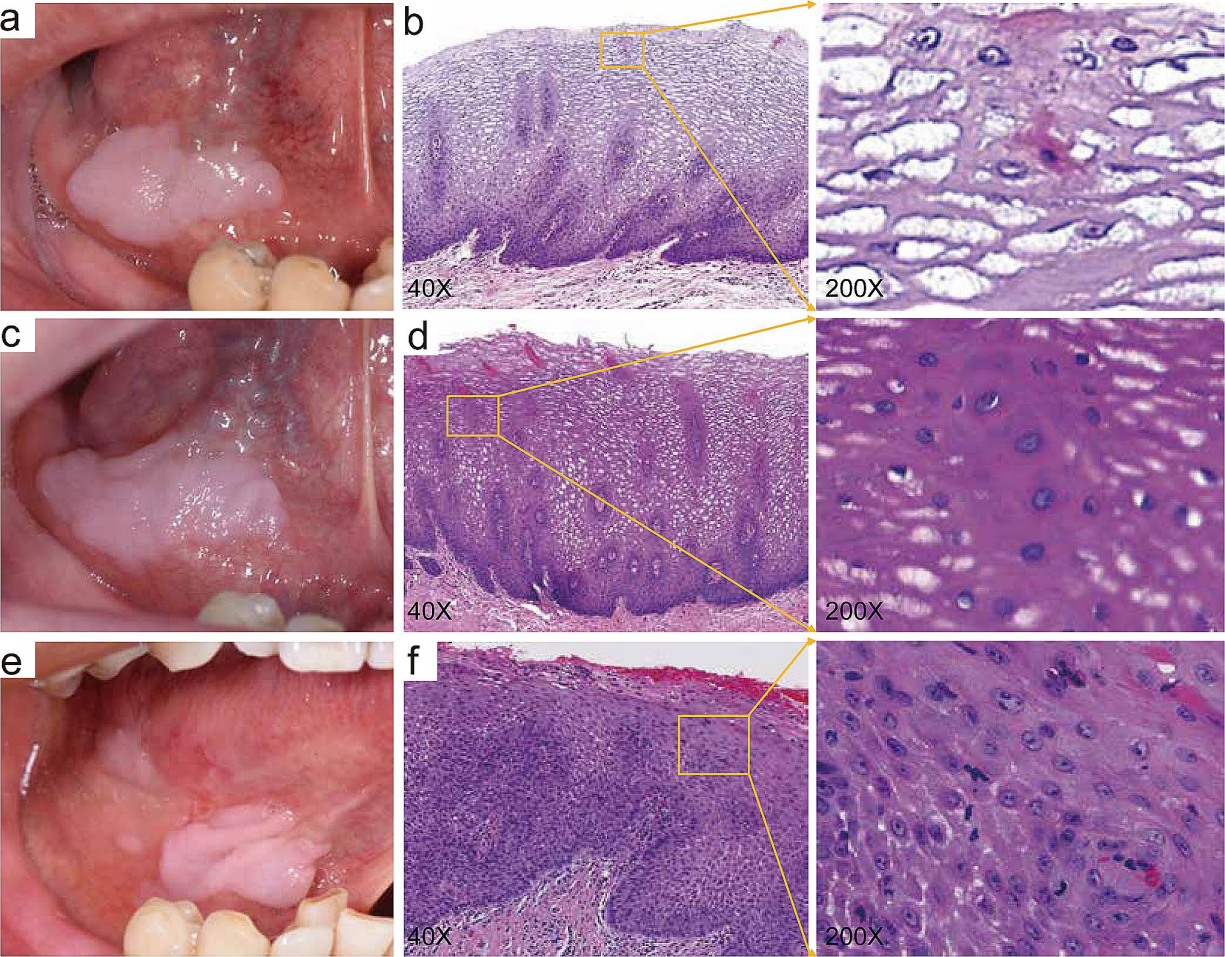
Clinical and pathological features of the case. (**a**) Initial clinical presentation showing a white lesion, diagnosed as White Sponge Nevus (WSN), on the floor of the mouth. (**b**) Histopathological examination from the first biopsy, demonstrating epithelial hyperplasia and thickening, along with vacuolar degeneration of spinous cells and pyknotic nuclei within the spinous layer. (**c**) Clinical image captured during the second visit, displaying an enlarged white lesion consistent with WSN. (**d**) Histopathological findings from the second biopsy, revealing pronounced vacuolar degeneration of spinous cells and thickening of the mucosal epithelium. (**e**) Clinical presentation during the third visit, showing both the white lesion and localized congestive atrophic changes. (**f**) Histopathological analysis from the third biopsy, indicating chronic mucosal inflammation and focal epithelial moderate-to-severe dysplasia, accompanied by excessive hypokeratinization and partial vacuolar degeneration of the epithelium. Note: All samples were stained with hematoxylin and eosin. Original magnification for panels (**b**), (**d**) and (**f**) is 200×

A 70-year-old male was referred for evaluation of a thick white lesion on the right floor of his mouth, present for three years (Fig. 1a). Histopathological examination revealed vacuolar degeneration of spinous cells and pyknotic nuclei, confirming a diagnosis of White Sponge Nevus (WSN) (Fig. 1b). The patient reported no associated pain or discomfort and had no similar lesions elsewhere on his body.

### Medical history and lifestyle

The patient had a 40-year history of smoking (≥ 20 cigarettes per day) but had ceased smoking for the past three years. He consumed alcohol occasionally, approximately once a month. Apart from hypertension, the patient reported no other significant medical history.

### Follow-up and progression

**Fig. 2:**
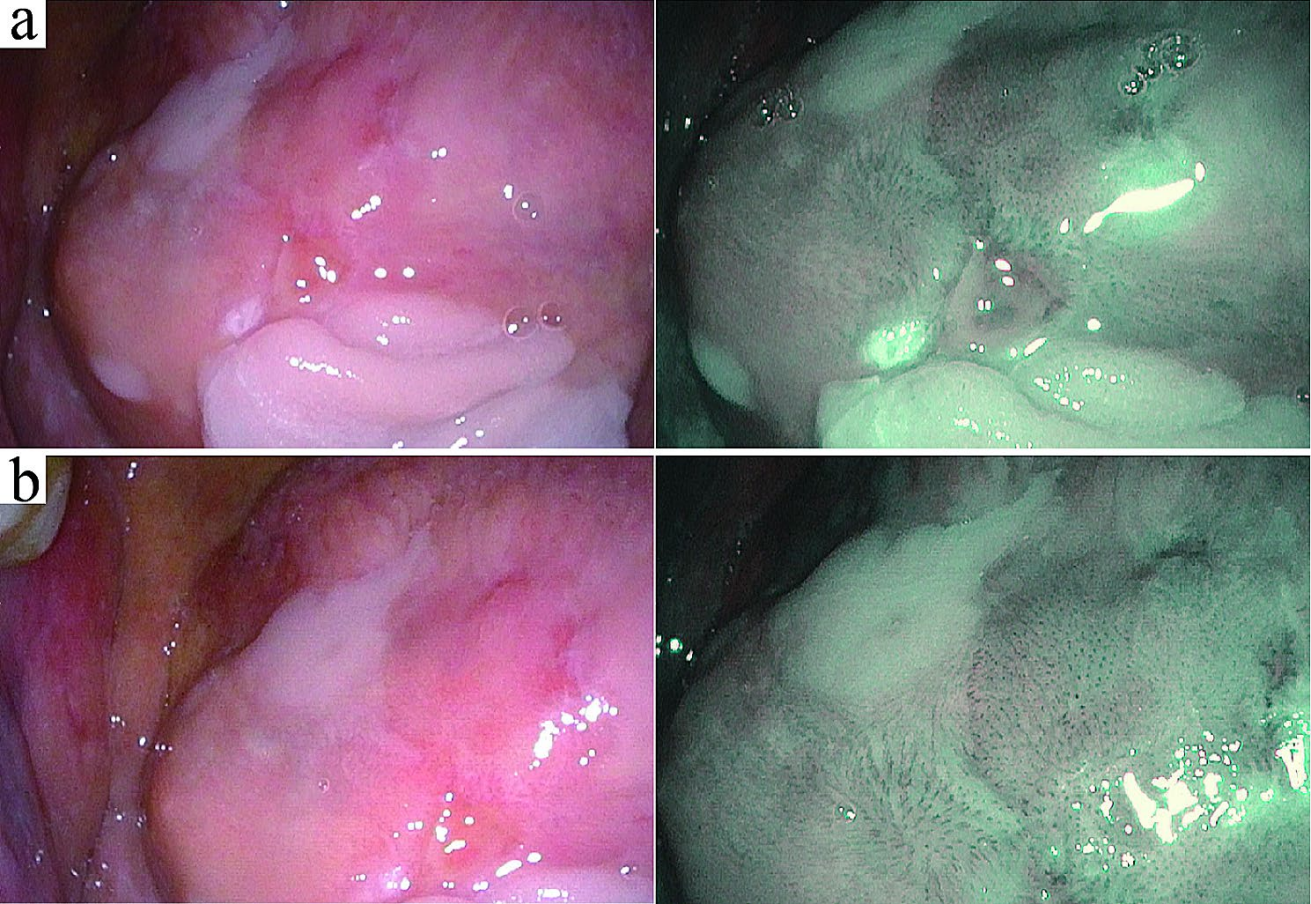
Narrowband imaging analysis of WSN lesions during the third follow-up visit. Panel (**a**) and panel (**b**) display narrowband imaging (NBI) results from distinct regions of the WSN lesion. Each panel illustrates paired images: the left image in each pair presents the conventional visualization of the lesion area, while the right image provides the corresponding NBI view. NBI analysis revealed local dilation of intrapapillary capillary loops and alterations in their arrangement patterns within the lesion area

**Fig. 3:**
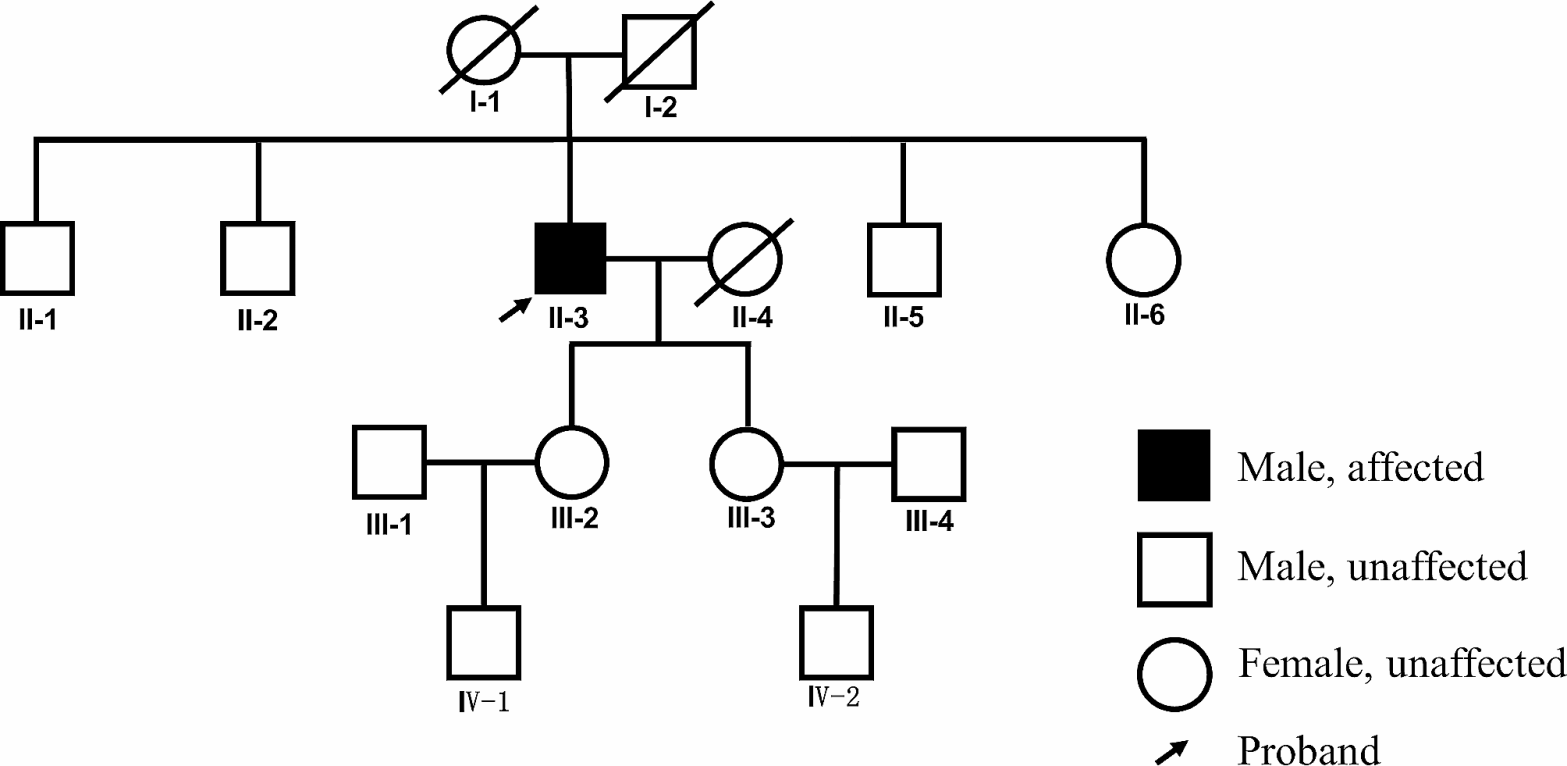
Family pedigree indicating keratin 4 (*KRT4*) mutation

At the six-month follow-up, a physical examination revealed an enlargement of the lesion, as depicted in Fig. 1c. Subsequent histopathological analysis from a second biopsy demonstrated features consistent with WSN and revealed mild chronic inflammatory cell infiltration within the lamina propria (Fig. 1d). During the 12-month follow-up, the patient reported symptoms of tongue numbness and mild pain while eating spicy foods or during tongue movements. Further examination showed localized congestion and atrophy in the affected area (Fig. 1e). To assist in the diagnosis and monitoring of potential malignant transformation, various noninvasive techniques were utilized [7, 8]. We used narrow-band imaging technology to assist in the diagnosis of WSN lesions [9], and the results revealed local dilation of intrapapillary capillary loops and alterations in their arrangement patterns within the lesion area (Fig. 2), suggesting malignant transformation of the epithelium. Therefore, a third biopsy was performed, confirming the presence of localized moderate-to-severe epithelial dysplasia (Fig. 1f). No similar lesions were observed in any immediate family members of the patient (Fig. 3). Following the confirmation of epithelial dysplasia, surgical resection of the lesion was undertaken. Subsequent monitoring over the next year revealed no recurrence of the lesion, suggesting successful management of this case.

### Genetic and molecular analysis

**Fig. 4:**
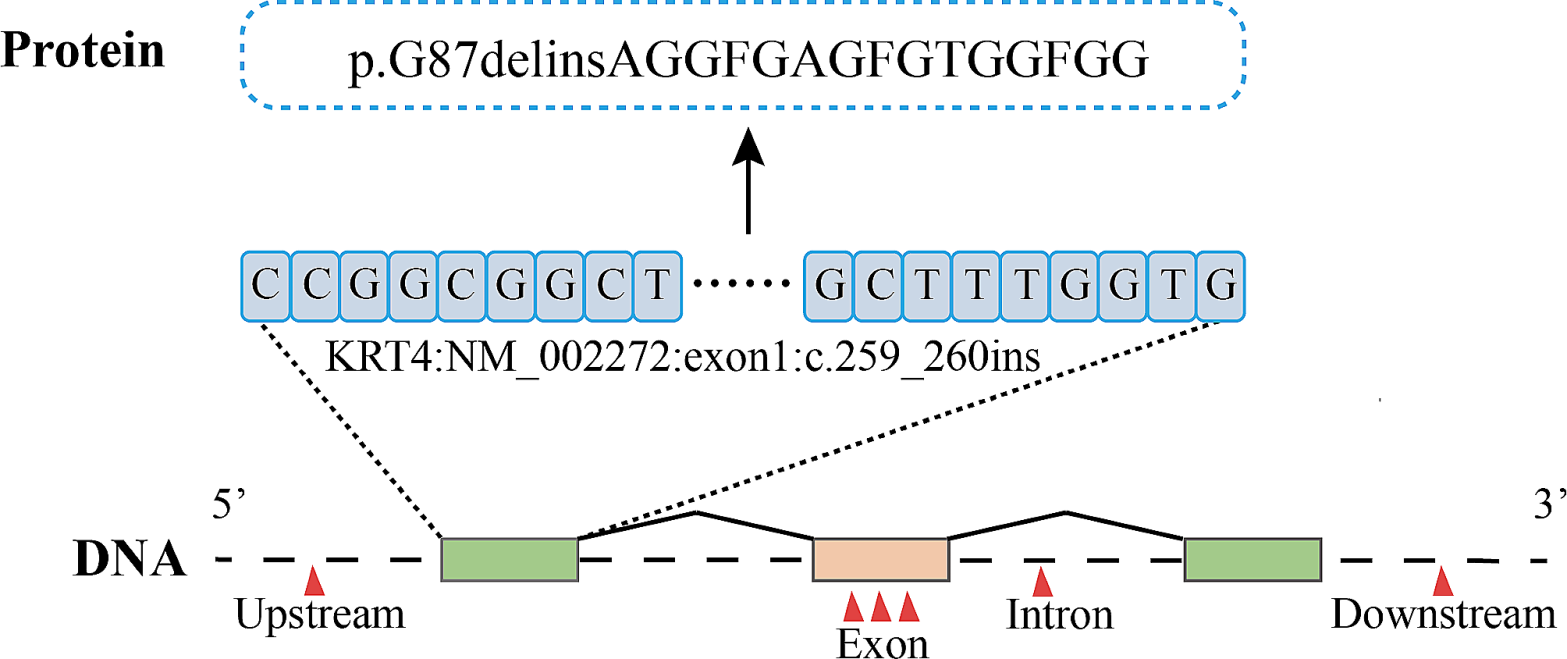
Mutation analysis. Schematic representation of the *KRT4* gene structure, with the novel variant indicated below

**Fig. 5:**
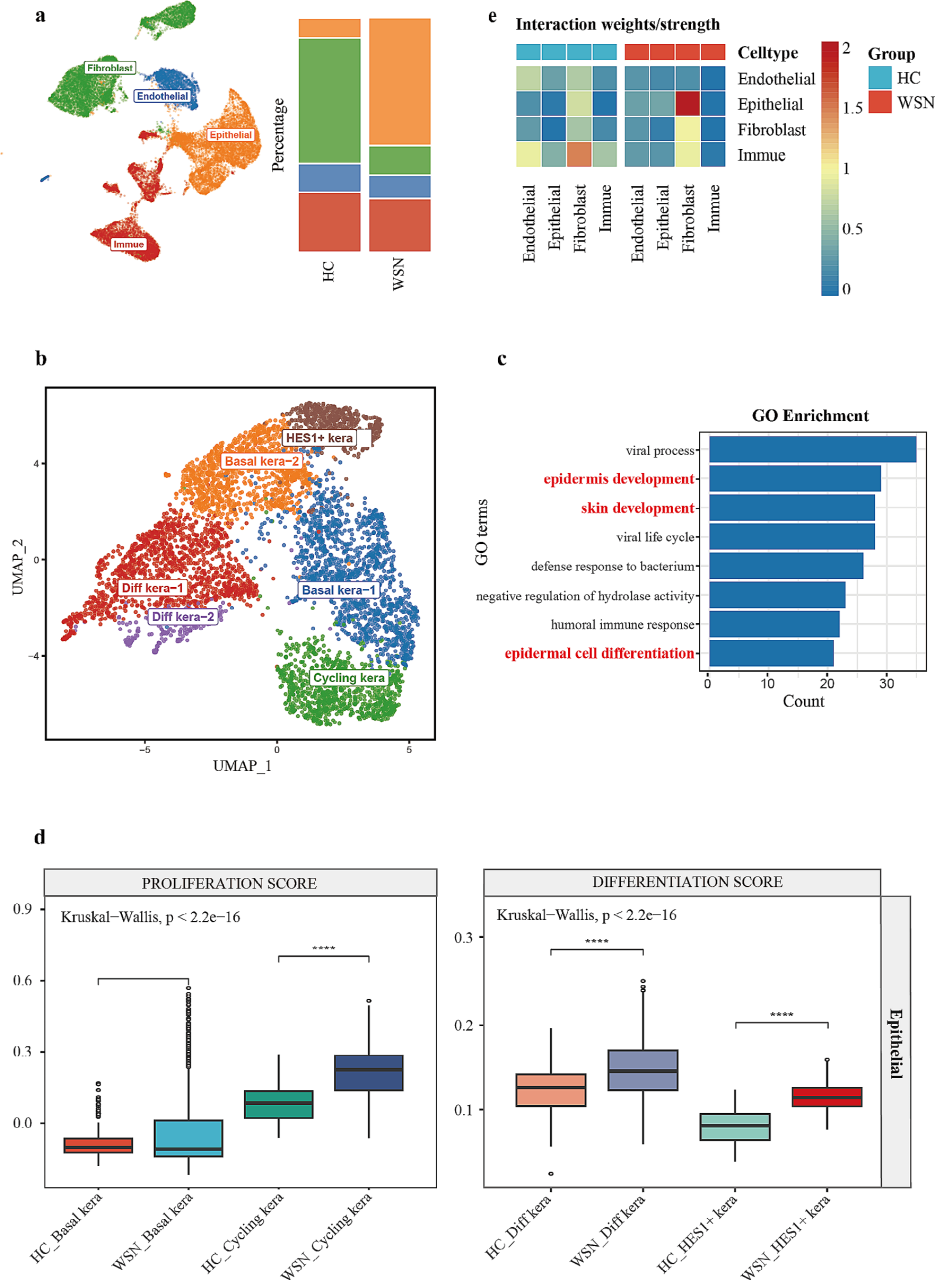
Dysregulated epithelial differentiation and proliferation in WSN. (**a**) UMAP (Uniform Manifold Approximation and Projection) representation illustrating the major cell types identified through single-cell RNA sequencing (left), accompanied by a bar graph depicting the relative proportions of each cell type (right). HC denotes healthy control; WSN represents white sponge nevus. (**b**) UMAP visualization of epithelial cell subtypes. (**c**) Bar plots highlighting biological processes enriched in the epithelium of WSN. (**d**) Box plots illustrating gene signature scores for epithelial cells, with each point representing a single cell. Statistical analysis was performed using the Kruskal-Wallis test with Benjamini-Hochberg correction. (**e**) Heatmap displaying cell-cell communication patterns among all identified cell types

Peripheral blood samples were collected from the patient (individual II-3, Fig. 3) and his healthy daughter (individual III-2, Fig. 3) for exome sequencing. A novel *KRT4* mutation was identified in the patient but not in his daughter (Fig. 4). To further investigate the impact of this novel *KRT4* mutation on the development of moderate-to-severe dysplasia in WSN, fresh lesion tissue was collected during the third biopsy for single-cell RNA sequencing. Healthy control data were obtained from the Human Protein Atlas (HPA). Initial analysis revealed a limited proportion of epithelial cells in healthy tissues (Fig. 5a). Subsequent re-clustering identified six epithelial subtypes: basal kera-1, basal kera-2, cycling kera, diff kera-1, diff kera-2, and HES1 + kera (Fig. 5b). Gene Ontology (GO) analysis indicated altered epidermal development activity (Fig. 5c). Furthermore, basal and cycling keratinocytes in WSN exhibited higher proliferative ability, while differentiating keratinocytes showed enhanced differentiation (Fig. 5d). Notably, communication between epithelial cells and fibroblasts was significantly increased (Fig. 5e).

## Discussion and conclusions

In this study, we report a one-year follow-up case of WSN with malignant transformation. Exome sequencing revealed a novel insertion mutation in exon 1 of *KRT4*, leading to a deletion-insertion mutation affecting glycine residues. Single-cell RNA sequencing further demonstrated altered epithelial proliferation and differentiation in the lesion.

Traditionally, WSN has been considered a benign genetic disorder with no malignant potential [10]. However, recent case reports challenge this view. A 2022 study in *Oral Oncology* found that the histomorphological features of oral squamous cell carcinoma (OSCC) closely resembled WSN, suggesting a potential malignant transformation [4, 11]. Additionally, Haseth et al. reported that 2 of 12 patients in a four-generation family with WSN developed OSCC [5]. Similarly, Downham et al. described a 59-year-old woman diagnosed with WSN who developed OSCC two years post-diagnosis [6]. This case report uniquely documents the complete progression of a WSN lesion from benign status to moderate-to-severe epithelial dysplasia within the same lesion site over a one-year observational period. This detailed account provides direct, sequential visual and histological evidence of the transformation process, illustrating the progression at regular intervals. Furthermore, our genetic analysis adds a significant dimension to the existing literature on WSN. By identifying a novel *KRT4* gene mutation associated with this case of WSN, we provide evidence that not all genetic mutations linked to WSN are benign. Contrary to mutations that have been previously reported to only lead to benign presentations, the mutation identified in this study appears to significantly increase the risk of malignant transformation. In light of these findings, we advocate for regular clinical follow-up to monitor WSN lesions, especially in patients with known risk factors such as a history of smoking, alcohol consumption, or personal or familial tumor history. This is particularly crucial for lesions located in high-risk areas of the oral mucosa, where early detection and timely intervention can potentially mitigate the progression to malignancy.

**Fig. 6:**
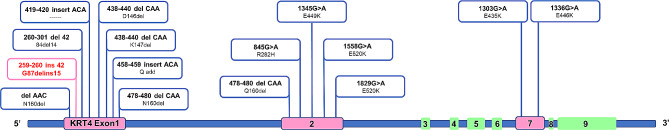
Summary of pathogenic mutations in the *KRT4* associated with WSN. The novel *KRT4* mutation identified in this study is highlighted within a red box

Previous research has established that WSN is commonly caused by pathogenic mutations in either *KRT4* or *KRT13* [6, 12]. Figure 6 presents a summary of case reports involving *KRT4* mutations in WSN. In the present case, exome sequencing identified a novel mutation in *KRT4* (exon 1:c.259_260insCCGGCGGCTTCGGAGCTGGTTTCGGCACTGGTGGCTTTGGTG), leading to a deletion-insertion amino acid mutation (p.G87delinsAGGFGAGFGTGGFGG).

This discovery broadens the known genetic spectrum of *KRT4* mutations associated with WSN. The primary roles of KRT include maintaining cellular integrity, facilitating intercellular adhesion, and participating in intracellular signal transmission, material transport, and cell differentiation [13, 14]. Altered expression patterns of KRT during and post-malignant transformation have been reported to influence various signaling pathways implicated in tumor progression [15]. Given these findings, we hypothesize that the novel *KRT4* mutation identified in this study may be a critical factor in the malignant transformation of WSN.

Single-cell RNA sequencing in our study indicated altered epithelial proliferation and differentiation, potentially contributing to epithelial dysplasia [15]. Literature also suggests that signaling pathways between epithelial cells and fibroblasts may promote esophageal squamous cell carcinoma [16]. Our data revealed enhanced communication between these cell types, warranting further investigation into the specific signaling pathways involved in WSN’s potential malignant transformation.

In conclusion, this case report significantly broadens the known spectrum of genetic mutations associated with WSN by documenting a novel mutation in the *KRT4* gene. Our findings elucidate the role of this mutation in promoting epithelial proliferation, differentiation, and enhanced intercellular communication, thereby advancing our understanding of the molecular mechanisms underlying WSN. These insights have crucial clinical implications for both the diagnosis and therapeutic management of WSN, suggesting that specific genetic profiles may require distinct clinical approaches.

However, this study does have its limitations. The single-case nature limits the generalizability of our findings, while the rarity of WSN complicates the accumulation of a large sample size. Additionally, follow-up challenges due to patient noncompliance and potential biases inherent in observational case studies further constrain our conclusions. Recognizing these limitations, we emphasize the need for future research to encompass a wider array of cases and employ a more systematic approach to follow-up. This will enhance the robustness of the findings and improve their applicability to a broader patient population.

Moreover, there is a pressing need for advancements in genetic and molecular diagnostic tools, which could enable more comprehensive and precise studies of malignant potential. Continued improvements in patient follow-up and detailed observation of oral lesions over extended periods will be crucial in uncovering more about the disease’s progression and potential malignancy.

Moving forward, our focus will remain on enhancing the longitudinal monitoring of WSN patients and adapting our clinical strategies based on evolving insights into the genetic and phenotypic characteristics of the disease.

## Data Availability

The datasets analyzed during the current study are available from the corresponding author on reasonable request.

## Abbreviations

WSN: white sponge nevus
KRT4: keratin 4
HPA: Human Protein Atlas
GO: Gene Ontology
OSCC: oral squamous cell carcinoma
